# Remnant cholesterol and the risk of cardiovascular disease in type 2 diabetes: a nationwide longitudinal cohort study

**DOI:** 10.1186/s12933-022-01667-6

**Published:** 2022-11-02

**Authors:** Ji Hye Huh, Kyung-do Han, Yun Kyung Cho, Eun Roh, Jun Goo Kang, Seong Jin Lee, Sung-Hee Ihm

**Affiliations:** 1 Department of Internal Medicine, Hallym University College of Medicine, Chuncheon, South Korea; 2 Department of Statistics and Actuarial Science, Soongsil University, Seoul, South Korea; 3 Department of Internal Medicine, Asan Medical Center, University of Ulsan College of Medicine, Seoul, Republic of Korea; 4 Asan Diabetes Center, Asan Medical Center, Seoul, Republic of Korea; 5 Division of Endocrinology and Metabolism, Hallym University College of Medicine, 24252 Hallymdaehak-Gil Chuncheon-si, Gangwon-do South Korea

**Keywords:** Type 2 diabetes, Remnant cholesterol, Cardiovascular disease, Triglyceride

## Abstract

**Background:**

Elevated remnant cholesterol (remnant-C) is considered a risk factor for cardiovascular disease (CVD); however, whether this notion applies to the East Asian population with type 2 diabetes (T2D) has not been established. This study investigated the association between remnant-C concentrations and the risk of CVD in Korean patients with T2D.

**Methods:**

By using the Korean National Health Insurance Service database, 1,956,452 patients with T2D and without atherosclerotic CVD who underwent regular health checks between 2009 and 2012 were included. Cox regression analyses were conducted to assess the association between remnant-C concentrations and incident CVD comprising myocardial infarction (MI) and ischemic stroke.

**Results:**

In total, 50,120 (2.56%) cases of MI and 73,231 (3.74%) cases of ischemic strokes occurred during a median follow-up of 8.1 years. The adjusted hazard ratios for MI and stroke in the highest remnant-C quartile were 1.281 (95% confidence interval [CIs], 1.249–1.314) for MI and 1.22 (1.195–1.247) for ischemic stroke, compared to those in the lowest quartiles. The results were similar, based on stratified analysis by age, sex, use of statin or fibrate, and levels of other cholesterol. The increased risk of CVD in the highest remnant-C quartile was profound in patients who had a longer T2D duration. A remnant-C concentration ≥ 30 mg/dL differentiated patients who were at a higher risk of CVD, compared to patients with a lower concentrations, regardless of whether LDL-C levels were or were not on target at ≤ 100 mg/dL.

**Conclusion:**

In Korean patients with T2D, remnant-C was associated with CVD, independent of the LDL-C level or other conventional CVD risk factors. Our finding confirmed evidence of the causal role of remnant-C on CVD, as a residual risk of CVD, in East Asian patients with T2D.

**Supplementary Information:**

The online version contains supplementary material available at 10.1186/s12933-022-01667-6.

## Background

Atherosclerotic cardiovascular disease (ASCVD) remains a leading cause of morbidity and mortality for patients with type 2 diabetes (T2D) [1]. However, only few people with T2D have optimal risk factor control and many patients with T2D still have a high residual risk [2]. Therefore, numerous efforts have been made to identify these residual risks for cardiovascular disease (CVD) in patients with T2D.

Atherogenic dyslipidemia frequently occurs in T2D. It is characterized by low levels of high-density lipoprotein cholesterol (HDL-C), often associated with elevated triglyceride (TG) levels (contained in very-low-density lipoproteins, intermediate-density lipoproteins, and their remnants—collectively called “triglyceride-rich lipoproteins” [“TRLs”]) [3]. These traits have been associated with increased CVD risk [4]. It is considered a residual risk factor in T2D [5–7].

Remnant cholesterol (remnant-C) is the cholesterol content of TRLs [8]. TRLs are more abundant, larger, and carry more cholesterol than do LDLs in atherogenic dyslipidemia. Previous studies have shown that remnant-C was associated with the risk of myocardial infarction (MI), stroke, and all-cause mortality in the general population [9–11]. A recent study demonstrated that remnant-C predicts ASCVD beyond low-density lipoprotein cholesterol (LDL-C) levels, but the investigators suggested that TG or remnant cholesterol-associated ASCVD risk is proportional to LDL-C [9]. Limited information about this issue is available among patients with only T2D who have a higher chance of atherogenic dyslipidemia. Therefore, the aim of the present study was to evaluate the association between remnant-C and the risk of CVD in T2D patients without a known history of CVD in a large pooled primary prevention cohort using the National Health Insurance Service (NHIS) health checkup data. We also investigated the risk of CVD associated with remnant-C concentrations, independent of LDL-C concentrations.

## Methods

### The NHIS database and NHIS Health Checkup Program

We used data from the National Health Insurance Service-Health Screening Cohort (NHIS-HEALS), which is provided by the Republic of Korea. In Korea, 97% of the population is obliged to enroll in this program [12]. The NHIS manages a biennial health checkup program for all insured South Koreans > 40 years of age and an annual checkup that is recommended for employee subscribers who are ≥ 20 years of age. The NHIS health checkup programs include anthropometric measurements; hearing and visual acuity checks; laboratory tests; past family, medical, and surgical history; and social history. Hospitals perform health checkups after being certified by the NHIS, which also regularly qualifies trained examiners. The NHIS-HEALS collects patients’ demographic data, such as region, age, sex, medical utilization/transaction information, claims and deduction data, and insurers’ payment coverage. The NHIS database has been described in detail in previous studies [13].

### Study population

In the NHIS Health Checkup database from 2002 to 2018, we selected adults aged 20 years and older who had a diagnosis of T2D and underwent health examinations from 2009 to 2012 (*n* = 2,746,079). We followed up with the individuals until the date of incident CVD or until December 31, 2018. We excluded people who is younger than 20 years old (n = 441) and who had received a diagnosis of MI or ischemic stroke before the index date of January 1, 2013 (n = 382,404). To minimize the influence of possible “reverse causation,” we excluded individuals who were developed MI, stroke, or CV deaths within 1 year after the baseline measurements (n = 35,063). Then, we also excluded patients with a TG level ≥ 300 mg/dL (n = 287,482) and who had any missing variables for measurement of remnant cholesterol (n = 84,237). Finally, 1,956,452 total participants were included in this study. (men = 1,159,932 and women = 796,520), and the median observational time was 8.15 (7.02–9.08) years (Figure S1). This study was approved by the Institutional Review Board of the Hallym University Sacred Heart Hospital (An-yang Si, South Korea; IRB No. HALLYM 2020-05-001), and permission was granted to use the NHIS Health Checkup data (NHIS-REQ000042107-001). The requirement for written informed consent was waived in the present study because data in the NHIS database are anonymized in adherence to strict confidentiality guidelines.

### Data collection

Detailed information on the individuals’ demographics and lifestyles was obtained through standardized self-reporting questionnaires. Income level was dichotomized at the lower 20%. Smoking status was classified as “nonsmoker,” “ex-smoker,” or “current smoker.” Alcohol drinking was categorized as 0 g/day (“none”), 30 g/day (“mild”), or ≥ 30 g/day (“heavy”). Regular exercise was defined as vigorous-intensity physical activity, performed at least three times per week, or as moderate-intensity physical activity, performed at least five times per week [14]. The health examinations provided by NHIS include anthropometric and laboratory measurements. Height, weight, and waist circumference (WC) were measured, and body mass index (BMI) was calculated by dividing weight (kg) by height (m) squared.

### Measurement of the lipid profile

Blood samples for the measurement of the lipid profile and glucose were drawn after an overnight fast. Lipid profiles, which included the levels of total cholesterol, LDL-C, high‐density lipoprotein cholesterol (HDL‐C), and TGs, were measured by using an enzymatic method. Quality control of laboratory tests was conducted by using the procedures of the Korean Association of Laboratory Quality Control [15]. Remnant-C level was estimated as the total cholesterol level minus the LDL-C level minus the HDL-C level. No established method exists to measure the remnant-C level, although previous studies have frequently used this equation because remnant-C levels can be easily obtained from the standard lipid profile [8]. The non-HDL-C level was calculated as the total cholesterol level minus the HDL-C level [10].

### Definition of comorbidities

All participants were categorized into three groups, based on their glycemic status: newly diagnosed T2D, T2D < 5 years, and T2D ≥ 5 years [16]. Patients with newly diagnosed T2D were diagnosed with T2D at the time of national health examinations in 2009–2012. Newly diagnosed T2D was defined as a fasting plasma glucose level ≥ 126 mg/dL during health examinations or at least one claim per year for the prescription of hypoglycemic drugs under International Classification of Disease (ICD-10) codes E11–14 in an outpatient or inpatient setting and a prescription for at least one antidiabetic drug at any time over 1 year to exclude prediabetic or nondiabetic individuals. Patients with T2D < 5 years were diagnosed with T2D within 5 years of the date of the health checkup in 2009–2012. Thus, these patients had been newly diagnosed with T2D from 2004 to 2008. Patients with T2D ≥ 5 years were diagnosed with T2D 5 years before 2009. Thus, these patients had been newly diagnosed with T2D before 2003.

Hypertension was defined as a blood pressure ≥ 140/90 mmHg or at least one claim per year for antihypertensive medication prescription under the *International Classification of Disease, 10th Revision* (ICD-10) codes I10–I15. “Dyslipidemia” was defined as a total cholesterol level ≥ 240 mg/dL or at least one claim per year for the prescription of lipid-lowering agents under ICD-10 code E78 [16]. “Chronic kidney disease” was defined as a glomerular filtration rate of < 60 mL/min/1.73 m^2^ and was based on a combination of ICD-10 codes (i.e., N18-19, Z49, Z94.0, and Z99.2). “Metabolic syndrome” was defined, based on the modified criteria of the National Cholesterol Education Program Adult Treatment Panel III (NCEP-ATP III) [17]. “Obesity” was defined as a BMI ≥ 25 kg/m^2^, using the Asia–Pacific criteria of the World Health Organization guidelines [17]. We defined a statin user as a person who had been prescribed statins during 2009–2012 and a fibrate user as a person who had been prescribed fibrates during 2009–2012.

### Outcome ascertainment

The primary endpoints of the study were newly developed MI or stroke. The diagnosis was based on the ICD-10 codes. MI was determined, based on the recording of ICD-10 code I21 or I22 during hospitalization for ≥ 4 days or the recording of these codes at least twice [18]. Ischemic stroke was determined, based on the recording of the ICD-10 code I63 or I64 during hospitalization for ≥ 4 days with claims for brain magnetic resonance imaging or brain computerized tomography [13]. The study participants were censored at date of the occurrence of MI or stroke, or end of follow-up (December 31, 2018), whichever came first.

### Statistical analyses

The baseline characteristics of the participants are expressed as the mean ± the standard deviation for continuous variables and as the number (percentage) for categorical variables. Values between groups were compared using the independent *t*-test for continuous variables and the chi-squared test for categorical variables. To compare each group, we performed one-way analyses of variance (ANOVA), and Chi square test, as appropriate. The incidence rates (IRs) of MI and ischemic stroke were calculated by dividing the number of events by 1000 person-years. Cox proportional hazard analysis was used to evaluate the association of remnant-C with MI and ischemic stroke and to calculate the hazard ratios (HRs) and 95% confidence intervals (CIs). Multivariable fully adjusted Cox proportional hazards models were applied. We assessed PH assumption using Schoenfeld residuals analysis and Log-log plot in our cox proportional hazard model. In the present study, subgroup analyses were also conducted using multivariable Cox proportional hazard models stratified by potential modifiable clinical factors through stratified analysis and interaction testing using the likelihood ratio test. Values of P < 0.05 were statistically significant. Statistical analysis was conducted using SAS 9.4 (SAS Institute Inc., Cary, NC, USA) and R 3.1.0 (R Foundation for Statistical Computing, Vienna, Austria).

## Results

### Baseline characteristics of the study population

The baseline characteristics of the study participants are presented in remnant-C quartiles in Table 1. Participants in the higher remnant-C quartiles were more likely to be male and younger than participants in the lower remnant-C quartiles, and they had worse metabolic traits. They had a higher proportion of current smokers and heavy alcohol drinkers, and they were more likely to exercise less regularly. Hypertension and dyslipidemia were more prevalent in patients in the higher remnant-C quartiles. Participants in the higher remnant-C quartiles had a low prevalence of longer duration of T2D (i.e., ≥ 5 years) and a lower proportion of insulin users.

**Table 1:** Baseline characteristics of patients with type 2 diabetes according to the remnant cholesterol quartiles

	Remnant cholesterol				
	**Q1**	**Q2**	**Q3**	**Q4**	**p-value**
Number	512,768	442,924	521,435	479,325	
Age (years)	56.92 ± 12.42	57.54 ± 12.06	56.83 ± 12.01	55.04 ± 12.01	< 0.0001
Male (%)	283,065(55.2)	250,503(56.56)	311,079(59.66)	315,285(65.78)	< 0.0001
Body mass index (kg/m^2^)	23.96 ± 4.5	24.87 ± 3.41	25.34 ± 3.37	25.73 ± 3.33	< 0.0001
Waist circumference (cm)	82.1 ± 9.13	84.66 ± 8.76	86.01 ± 8.47	87.14 ± 8.38	< 0.0001
Systolic blood pressure (mmHg)	126.24 ± 15.62	128.27 ± 15.63	129.29 ± 15.64	130.33 ± 15.64	< 0.0001
Diastolic blood pressure (mmHg)	77.03 ± 10.03	78.5 ± 10.03	79.41 ± 10.12	80.52 ± 10.21	< 0.0001
Fasting blood glucose (mg/dl)	139.02 ± 42.73	141.92 ± 43.77	145.07 ± 45.38	150.62 ± 48.52	< 0.0001
Total cholesterol (mg/dl)	181.64 ± 39.82	192.08 ± 41.46	199.79 ± 42.5	208.93 ± 45.25	< 0.0001
HDL cholesterol (mg/dl)	56.88 ± 14.59	53.16 ± 13	50.61 ± 12.37	48.07 ± 11.89	< 0.0001
LDL cholesterol (mg/dl)	110.08 ± 37.07	115.98 ± 39.28	117.65 ± 40.41	113.86 ± 42.99	< 0.0001
Non-HDL cholesterol (mg/dL)	124.75 ± 37.5	138.92 ± 39.38	149.19 ± 40.54	160.86 ± 43.44	< 0.0001
Remnant cholesterol (mg/dL)	14.67 ± 3.34	22.94 ± 1.99	31.54 ± 3.13	47 ± 11.04	< 0.0001
Triglyceride (mg/dL)	71.81 (71.76–71.87)	113.32 (113.28-113.36)	155.53 (155.47-155.58)	227.91 (227.81-228.01)	< 0.0001
Smoking status					< 0.0001
Non-smoker	318,338(62.08)	261,330(59)	287,071(55.05)	234,076(48.83)	
Ex-smoker	92,129(17.97)	78,897(17.81)	94,861(18.19)	89,315(18.63)	
Current smoker	102,301(19.95)	102,697(23.19)	139,503(26.75)	155,934(32.53)	
Alcohol drinking					< 0.0001
Non	314,134(61.26)	265,057(59.84)	294,255(56.43)	238,044(49.66)	
Mild	162,645(31.72)	141,544(31.96)	175,498(33.66)	178,612(37.26)	
Heavy	35,989(7.02)	36,323(8.2)	51,682(9.91)	62,669(13.07)	
Regular exercise	123,172(24.02)	95,612(21.59)	104,569(20.05)	89,334(18.64)	< 0.0001
Income, low 20%	109,868(21.43)	93,352(21.08)	109,562(21.01)	100,378(20.94)	< 0.0001
Hypertension	244,395(47.66)	237,028(53.51)	289,147(55.45)	266,913(55.69)	< 0.0001
Dyslipidemia	159,422(31.09)	162,658(36.72)	207,102(39.72)	210,736(43.97)	< 0.0001
Chronic kidney disease	43,907(8.56)	45,309(10.23)	55,313(10.61)	49,440(10.31)	< 0.0001
Duration of diabetes ≥ 5years	163,716(31.93)	134,867(30.45)	147,739(28.33)	119,014(24.83)	< 0.0001
Statin user	122,853(23.96)	117,463(26.52)	139,006(26.66)	124,018(25.87)	< 0.0001
Fibrate user	9806(1.91)	9677(2.18)	12,358(2.37)	16,271(3.39)	< 0.0001
Insulin use	47,708(9.3)	34,637(7.82)	36,859(7.07)	29,225(6.1)	< 0.0001

Data are expressed as the mean ± SD, median (25–75%), or n (%)

BP, blood pressure; eGFR, estimated glomerular filtration rate; HDL, high-density lipoprotein; LDL, low-density lipoprotein; TC, total cholesterol; TG, triglyceride; OHA, oral anti-hypoglycemic agents

P-values for the trend were < 0.0001 for all variables because of the large size of the study population

### Baseline lipid concentrations and major cardiovascular events

During the median (interquartile range) follow-up duration of 8.15 (7.02–9.08) years, 123,351 individuals experienced the new development of MI or stroke (50,120 cases of MI and 73,231 cases of stroke). The association between lipid concentrations and risk of MI and stroke is presented in Table S1. The serum concentrations of LDL-C, TG, and non-HDL-C were associated with a 1.4%, 1.5%, and 1.6% higher risk of MI per every 10 mg/dL increase, respectively. The serum concentrations of HDL-C were associated with a 2% lower risk of MI per every 5 mg/dL increase, whereas the concentrations of remnant-C was associated with a 3.4% higher risk of MI per 10 mg/dL increase. The serum concentrations of LDL-C, TG, and non-HDL-C were likewise associated with a 1.1%, 1.2%, and 1.2% higher risk of stroke per every 10 mg/dL increase, respectively. The serum concentration of HDL-C was associated with a 1.4% lower risk of stroke per every 5 mg/dL increase and the concentration of remnant-C was associated with a 3.2% higher risk of stroke per 10 mg/dL increase. We further examined the association between the remnant-C quartile and the risk of MI and ischemic stroke (Table 2). The incidence of MI was particularly high in participants in the upper remnant-C quartiles, compared to the lowest quartile, even after adjusting for the mediators of potential effects of remnant-C levels such as body mass index, drinking status, use of lipid-lowering agents (e.g., statin or fibrate) and fasting blood glucose levels. In particular, the highest remnant-C quartile was associated with a 28.1% higher risk of MI and 22% higher risk of stroke, even after adjusting for mediators of the potential effects of remnant-C levels [a HR (95% CI) of MI in the fourth remnant-C quartile was 1.281 (1.249–1.314), and the HR (95% CI) of stroke in the fourth remnant-C quartile was 1.22 (1.195–1.247)].

**Table 2:** Risk of myocardial infarction and stroke across quartiles of remnant cholesterol quartiles

	Events	Duration	Incident rate (1000 person-year)	Unadjusted model HRs (95% CI)	Age, sex adjusted model HRs (95%CI)	Fully adjusted model* HRs (95%CI)
**Myocardial infarction**						
**Remnant cholesterol (Q1)**	17,143	3485371.99	4.919	Reference	1(Ref.)	Reference
**Remnant cholesterol (Q2)**	16,885	3020245.84	5.591	1.128(1.099,1.157)	1.103(1.075,1.132)	1.083(1.055,1.112)
**Remnant cholesterol (Q3)**	20,563	3563850.44	5.770	1.199(1.17,1.229)	1.21(1.181,1.241)	1.175(1.146,1.205)
**Remnant cholesterol (Q4)**	18,640	3287793.49	5.669	1.224(1.193,1.254)	1.333(1.3,1.367)	1.281(1.249,1.314)
**Stroke**						
**Remnant cholesterol (Q1)**	17,143	3485371.99	4.919	1(Ref.)	1(Ref.)	Reference
**Remnant cholesterol (Q2)**	16,885	3020245.84	5.591	1.136(1.112,1.16)	1.108(1.084,1.131)	1.083(1.06,1.106)
**Remnant cholesterol (Q3)**	20,563	3563850.44	5.770	1.172(1.149,1.196)	1.194(1.17,1.218)	1.147(1.124,1.171)
**Remnant cholesterol (Q4)**	18,640	3287793.49	5.669	1.152(1.128,1.176)	1.3(1.273,1.327)	1.22(1.195,1.247)

*Adjusted model: Adjusted for age, sex, body mass index, smoking status, alcohol drinking status, regular exercise, low income, hypertension, statin use, fibrate use, fasting blood glucose and diabetes duration (≥ 5years)

*HRs*, hazard ratios

### Association between remnant-C level and the risk of CVD, based on the duration of diabetes

**Fig. 1:**
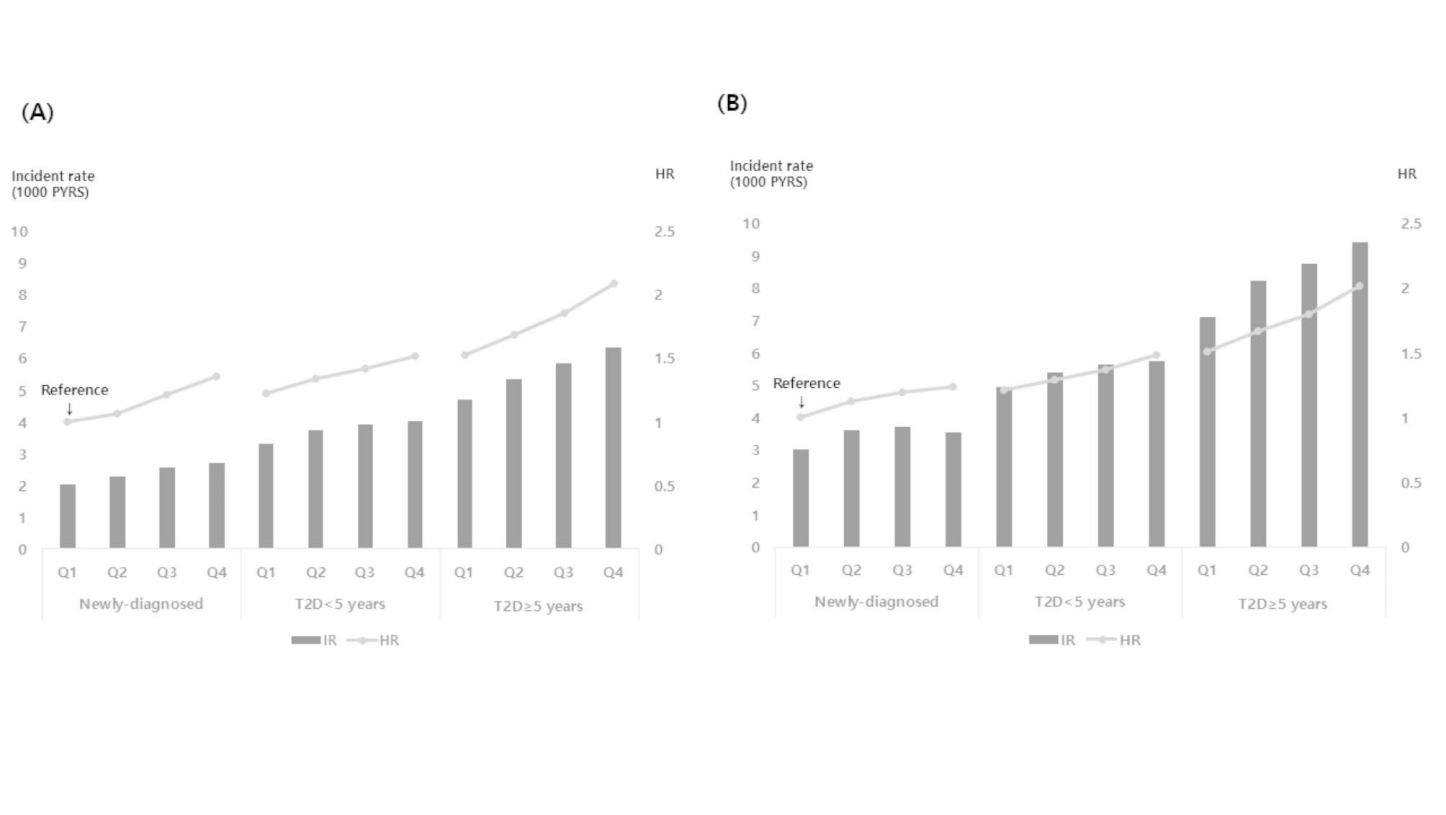
Incident rate of myocardial infarction (A) and stroke (B) per 1000 person-years (bar) and adjusted hazard ratios of myocardial infarction and stroke (line) by quartiles of remnant cholesterol according to diabetes status. * adjusted for age, sex, body mass index, smoking status, alcohol drinking status, regular exercise, low income, hypertension, statin treatment, and fibrate use T2D, type 2 diabetes; HR, hazard ratio; IR. incident rate; PYRS, person years

We examined the risk of MI and stroke, based on the remnant-C quartiles, stratified by the duration of T2D (Fig. 1). Among patients who had newly developed T2D, the IRs of MI and stroke tended to be incrementally higher in the upper remnant-C quartiles than in the lowest quartile However, IRs of stroke were highest in the third quartile of the newly developed T2D group. However, in the groups with T2D < 5 years or T2D ≥ 5 years, the IRs of MI and stroke were incrementally higher in the upper remnant-C quartiles than in the lowest quartile, and it increased with the progression of diabetes. This linear trend between remnant-C levels and incident CVD was more prominent in patients with a longer duration of T2D (i.e., ≥ 5 years). The risks of MI and stroke gradually increased with the progression of the diabetes status and increased remnant-C concentrations, using newly developed T2D with the lowest remnant-C quartile as the reference (for the fully adjusted HRs of MI in the highest remnant-C quartile: HR = 1.356 for newly diagnosed T2D, HR = 1.515 for T2D < 5 years, and HR = 2.085 for T2D ≥ 5 years; for the fully adjusted HRs of stroke in the highest remnant-C quartile: HR = 1.238 for newly diagnosed T2D, HR = 1.485 for T2D < 5 years, and HR = 2.016 for T2D ≥ 5 years). It indicates that a higher risk of CVD in higher remnant-C concentration was more profound in patients with a longer duration of T2D.

### Risk of CVD, based on the remnant-C quartile in the various subgroups

**Fig. 2:**
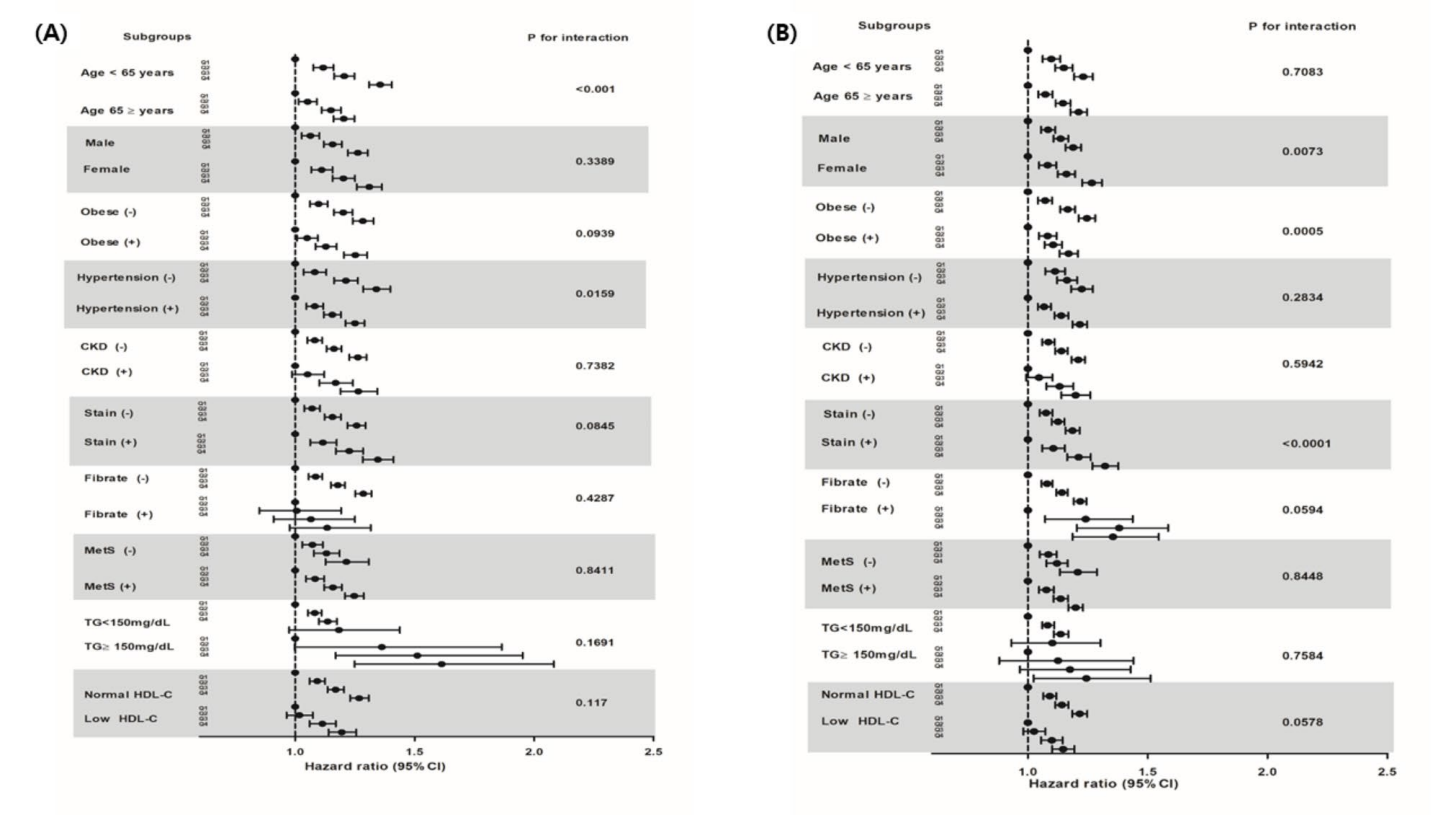
Hazard ratios and 95% confidence intervals of myocardial infarction (A) and ischemic stroke (B) according to the quartiles of the remnant cholesterol in subgroups. ^#^Adjusted model: Adjusted for age, sex, body mass index, smoking status, alcohol drinking status, regular exercise, low income, hypertension, statin use, fibrate use, duration of diabetes, and fasting blood glucose. CKD, chronic kidney disease; MetS, metabolic syndrome; TG, triglyceride; HDL-C; high density lipoprotein cholesterol; CI, confidence interval; HR, hazard ratio

We conducted subgroup analyses, stratified by age, sex, obesity status, absence or presence of hypertension, chronic kidney disease, metabolic syndrome, hypertriglyceridemia (TG, ≥ 150 mg/dL), low HDL-C (< 40 mg/dL in men and < 50 mg/dL in women), and use of statin or fibrate drugs (Fig. 2 and Table S2).

The higher risk of MI and stroke in the upper remnant-C quartiles was sustained consistently across all subgroups of patients with T2D. The higher risk of MI and stroke in the upper remnant-C quartiles persisted notably, regardless of the TG or HDL-C level and the use of medication for dyslipidemia. The association between remnant-C level and MI risk was stronger in younger (i.e., < 65 years) patients with T2D, and in patients without hypertension. The association between the remnant-C level and ischemic stroke risk was stronger in women, statin users, and in patients without obesity or abdominal obesity.

### Contribution of the remnant-C level to the development of CVD, based on the LDL-C level

Based on definitions used in a previous study [10], we categorized our patients into four groups, using the combinations of each remnant-C and LDL-C cutoff level (Table 3). We found that 446,210 patients with T2D (i.e., 22.8% of the entire cohort) were at the target for both lipid values. However, 14.5% of patients were classified with a high remnant-C level and a target level of LDL-C. Even in patients with controlled LDL-C levels, high levels of remnant-C were associated with a 13.8% higher risk of MI and 10.1% risk of stroke, compared to patients with lower concentrations of remnant-C within the same LDL-C subgroup [the fully adjusted HR (95% CI) of MI was 1.138 (1.104–1.173); the fully adjusted HR of stroke was 1.101 (1.074–1.128) in the remnant-C/LDL-C [≥ 30/<100] group]. It indicates the residual lipid risk assessed by a remnant-C ≥ 30 mg/dl was associated with higher cardiovascular risk, regardless of LDL-C concentrations.

**Table 3:** Risk of myocardial infarction and stroke based on categories of low-density lipoprotein cholesterol and remnant cholesterol levels

	Number	Events	Duration	Incident rate (1000 person-year)	HR(95% C.I)			
					**Unadjusted model**	**Adjusted model** ^**#**^		
**Myocardial infarction**								
**Remnant-C/LDL-C < 30/<100**	446,210	10,484	3030322.48	3.460	1(Ref.)	1(Ref.)		
**Remnant-C/LDL-C < 30/≥100**	731,540	18,135	5041016.74	3.597	1.037(1.012,1.062)	1.146(1.119,1.175)		
**Remnant-C/LDL-C ≥ 30/<100**	283,465	7321	1952671.09	3.749	1.08(1.049,1.113)	1.138(1.104,1.173)		
**Remnant-C/LDL-C ≥ 30/≥100**	495,237	14,180	3414422.56	4.153	1.198(1.168,1.228)	1.375(1.34,1.411)		
**Stroke**								
**Remnant-C/LDL-C < 30/<100**	446,210	16,175	3011899.92	5.370	1(Ref.)	1(Ref.)		
**Remnant-C/LDL-C < 30/≥100**	731,540	26,657	5010460.41	5.320	0.99(0.971,1.009)	1.078(1.057,1.1)		
**Remnant-C/LDL-C ≥ 30/<100**	283,465	10,909	1940361.97	5.622	1.046(1.021,1.071)	1.101(1.074,1.128)		
**Remnant-C/LDL-C ≥ 30/≥100**	495,237	19,490	3394539.47	5.742	1.069(1.047,1.092)	1.23(1.204,1.257)		

^#^Adjusted model: Adjusted for ag, sex, body mass index, smoking status, alcohol drinking status, regular exercise, low income, hypertension, statin use, fibrate use, duration of diabetes, and fasting blood glucose

CI, confidence interval; HR, Hazard ratio

## Discussion

In this nationwide population-based cohort study, we investigated the association between remnant-C concentrations and the risk of CVD in Korean patients with T2D, who were followed for more than approximately 8.15 years, by using data from the Korean National Health Insurance Service database. We noted the following findings: (1) the remnant-C level was significantly associated with CVD events consisting of MI and stroke in T2D, regardless of whether lipid-lowering agents were or were not used; (2) elevated remnant-C levels were associated with the risk of incident CVD, independent of traditional CVD risk factors, including LDL-C concentrations, in patients with T2D; in addition, elevated remnant-C levels increased the risk of CVD, even in patients who were at the target range of LDL-C concentrations for primary prevention in T2D; and (3) the linear association between elevated remnant-C levels and the higher risk of ASCVD was more prominent in patients with a more advanced stage of T2D. These findings indicated that the remnant-C level may be a significant residual risk factor for CVD in East Asian patients with T2D.

The increased CVD risk associated with a high remnant-C level in T2D is due, in part, to diabetes-related atherogenic dyslipidemia, which is characterized by high circulating TG and/or cholesterol content within TRLs and low concentrations of HDL-C with relatively normal concentrations of LDL-C. In particular, the relationship between hypertriglyceridemia and the risk of CVD in patients with T2D remains inconclusive [19]. TG, per se, is unlikely to directly cause CVD [20]; however, Nordestgaard et al. [5] demonstrated that the harmful component in TRLs is cholesterol, not TG itself. Furthermore, a recent systematic review demonstrated that, in patients with T2D, an elevated TG concentration is not an independent marker for an increased risk of ASCVD events [21], and increasing HDL-C levels fail to reduce ASCVD [6, 7]. These findings suggest that TG is a just marker of elevated remnant-C levels. Therefore, an estimation of remnant-C level may be more important than the TG or HDL-C level itself in patients with T2D [3]. However, few data exist on the role of remnant-C in the primary prevention of CVD, especially in patients with T2D. Therefore, this study has clinical implications showing that elevated remnant-C concentrations may be an independent residual risk factor for patients with T2D, which may provide new information on the necessity of monitoring remnant-C for the primary prevention of ASCVD in T2D.

The strong association between remnant-C concentrations and CVD in T2D patients can be explained by the following mechanisms. Cholesterol is the major lipid that accumulates in atherosclerotic lesions. Remnant-C has a greater cholesterol content per particle than does LDL-C, perhaps making remnant-C even more atherogenic than LDL-C [2]. It also passes into the arterial wall and is taken up by macrophages and smooth muscle cells without modification, unlike LDL, which needs to be modified before uptake. However, in diabetes the catabolism of chylomicron-derived remnant-C particles is decreased, thereby resulting in the accumulation of remnant-C particles in plasma [22]. Some early studies in which radiolabeled chylomicrons were injected in diabetic rats, demonstrated the accumulation of cholesteryl ester, but not TG components, in plasma, which suggested that chylomicron-derived remnant-C accumulates in diabetes [22]. Another study similarly showed that the retention of chylomicron remnants by the arterial intima was associated with hyperglycemia in diabetic rabbits and rats [23]. Hence, the atherogenic effects of remnant-C in elevated glucose levels may explain the associations between remnant-C and CVD, especially in the advanced stage of T2D, as demonstrated in the present study.

We demonstrated that remnant-C was the major cholesterol fraction contributor to the development of MI and stroke in patients with T2D without a history of ASCVD. Participants in our study had nearly normal or slightly increased TG concentrations (TG < 300 mg/dL), and a considerable percentage of patients (approximately 25%) were taking statin drugs. We found that individuals with high remnant-C levels (> 30 mg/dL) had a greater risk of MI and ischemic stroke, regardless of whether their LDL-C level was or was not at optimal levels (< 100 mg/dL). Based on the current findings, increased concentrations of remnant-C contribute to atherogenic risk, even in patients with T2D, independent of TG or LDL-C concentrations and statin use. Moreover, an implication is that the treatment of residual risk, based on measurements of the remnant-C concentration, may be more beneficial than further reducing LDL-C concentrations in patients with T2D for primary prevention if they are already at the target LDL-C concentration by taking a statin drug. This finding is consistent with findings from previous studies which was conducted in specific ethnic and disease groups on a small scale. Castaner et al. [10] reported that high remnant-C levels among obese and overweight patients were associated with a 2.6 times higher risk of ASCVD, although LDL-C levels being in the optimal range, Cao et al. [30] also demonstrated that remnant-C was associated with a 62% higher risk of ASCVD in T2D patients with a history of coronary artery disease. The absolute values of HRs in the present study seemed to be relatively lower than the values reported in previous studies. This finding could be explained by the fact that our cohort comprised a primary prevention cohort and other clinical conditions and confounding factors associated with T2D may attenuate the association between remnant-C and CVD. Considered together, we suggest that our findings provide more concrete evidence that measuring plasma remnant-C levels may be clinically relevant in primary prevention to identify T2D patients who are at high risk of CVD.

By using subgroup analysis, we found that higher remnant-C levels were consistently associated with an increased risk of MI and stroke in all clinical subgroups. The risk of CVD associated with remnant-C was more prominent in patients who had a relatively lower CVD risk such as patients with a younger age, absence of hypertension, and women. The risk of CVD associated with remnant-C was more prominent in patients who had a relatively lower CVD risk such as patients with a younger age, absence of hypertension, and women. We are not able to explain why individuals at relatively lower risk of CVD are more susceptible to elevated remnant-C levels. We assume that as individuals with multiple CVD risk factors would be influenced by other strong CVD risk factors rather than remnant-C. For example, because the proportion of men who currently smoke was higher than women, the independent risk of remnant-C on CVD in men would be attenuated by smoking.

Of interest, the association between the remnant-C level and CVD risk was more prominent in statin-treated patients. This finding indicated that, even when treated with statins, patients with high remnant-C levels were at high risk of CVD. European dyslipidemia guidelines recommend lowering the LDL-C and apolipoprotein B (apo B) levels in high-risk patients such as patients with T2D [24]. Another recent study [25] has also shown that elevated non-HDL cholesterol and apo B levels, but not LDL-C levels, are associated with the residual risk of MI and overall mortality in statin users. Given the 1:1 association between apoB and atherogenic lipoprotein particles, apoB concentrations represent the number of the lipoprotein particles. While, remnant-C represents cholesterol contents carried by the apolipoprotein B-containing lipoprotein particles. Therefore, these two factors are closely associated and both of two factors can be used to assess CV risk in individuals. Our findings confirmed the evidence supporting that the remnant-C level explains the residual risk of ASCVD in patients with T2D. We also suggest that the remnant-C level should be considered for use as an additional prognostic lipid measure, along with LDL-C in patients with T2D, even if they were already taking antidyslipidemic agents.

The higher CVD risk with higher remnant-C concentrations was interestingly more profound in patients with a more advanced stage of T2D in this study. A longer duration of diabetes increases the risk of ASCVD or CV deaths in patients with T2D [26]. Furthermore, another study [27] demonstrated that high plasma residual cholesterol was overproduced in the insulin-resistant status, and it may have a key role in the etiology of coronary artery disease in patients with diabetes. Several in vitro and in vivo studies [28] have also suggested that the macrophage uptake of remnant-C in diabetic patients was correlated with the degree of glycemic control in patients with diabetes. We assume that factors associated with an advanced stage of T2D may act as mediating factors that enhance intimal permeability or the capture of remnant-C within the arterial intima in the process of atherosclerosis [29]. These facts indicate that remnant-C is additionally interacting with a worsening glycemic status and combining effect of these two factors consequently have a stronger atherogenic potential in patients with T2D. Therefore, evaluating the combined effect of high remnant-C levels and a worsening glycemic status in T2D may provide new insight into cardiovascular events and metabolic risk estimation.

This study has several strengths. First, it is the first study to examine the association between remnant-C and MI or stroke, based on the duration of T2D status, in the Korean population. Second, this study had a large sample size, consisting of > 2,500,000 patients with T2D, and used a well-validated national database. However, several limitations of this study should be addressed. First, potential known confounding variables were adjusted for in multivariable Cox regression analysis; however, we could not exclude all possible residual bias because we did not collect all data about metabolic parameters related to CVD or lifestyle factors. Moreover, because NHIS-HEALS did not measure glycated hemoglobin, a well validated surrogate marker for the degree of glycemic control, we were unable to assess the association between remnant-C and CVD stratified by glycemic control status based on glycated hemoglobin. The remnant-C level calculated in the current study may have over- or under-estimated its value, compared to direct measurements. However, the calculation of the remnant-C level is an affordable method that could provide valuable data for clinical management. Therefore, many recent studies [30] have measured remnant-C by a calculation, as in our study. Third, we did not consider the effects of anti-diabetic medications, which might have potential effects on the development of CVD ((e.g. sodium-glucose co-transporter-2 [SGLT-2] inhibitors or glucagon like peptide-1 receptor agonists (GLP-1 RA)). However, these kinds of anti-diabetic medications are not widely availabe in Korea due to reimbursement restriction [31]. Finally, the present study was observational, and the causal role of remnant-C on ASCVD risk should be determined in further studies.

## Conclusion

This large-scale cohort study, which had a long-term follow-up, showed that remnant-C was a risk factor for MI and stroke in patients with T2D without a history of ASCVD, independent of traditional risk factors and serum LDL-C concentrations. Furthermore, we demonstrated that the higher CVD risk in patients with a higher remnant-C concentration was more profound in patients with a longer duration of T2D. This finding indicated that determining remnant-C levels in patients with T2D would be useful in terms of cardiovascular risk stratification and future interventions.

## Electronic supplementary material

Below is the link to the electronic supplementary material.

Supplementary Material 1. Figure S1. Flowchart of study participants. Table S1. Association of baseline lipid values with incident myocardial infarction and stroke in patients with type 2 diabetes mellitus. Table S2. Hazard ratios and 95% confidence intervals of myocardial infarction (A) and ischemic stroke (B) according to the quartiles of the remnant cholesterol in subgroups.

## Data Availability

The datasets used and/or analyzed during the current study are available from the corresponding author on reasonable request.

## Abbreviations

ASCVD: Atherosclerotic cardiovascular disease
CI: Confidence interval
CVD: Cardiovascular disease
HDL-C: High-density lipoprotein cholesterol
HR: Hazard ratio
IR: Incidence rate
LDL: Low-density lipoprotein
MI: Myocardial infarction
NHIS: National Health Insurance Service
remnant-C: Remnant cholesterol
RLP: Remnant-like particle
T2D: Type 2 diabetes
TRL: Triglyceride-rich lipoprotein

## References

[1] Rawshani A, Rawshani A, Franzen S, Sattar N, Eliasson B, Svensson AM, Zethelius B, Miftaraj M, McGuire DK, Rosengren A (2018). Risk Factors, Mortality, and Cardiovascular Outcomes in Patients with Type 2 Diabetes. N Engl J Med.

[2] Joshi PH, Martin SS, Blumenthal RS (2015). The remnants of residual risk. J Am Coll Cardiol.

[3] Sandesara PB, Virani SS, Fazio S, Shapiro MD (2019). The Forgotten Lipids: Triglycerides, Remnant Cholesterol, and Atherosclerotic Cardiovascular Disease Risk. Endocr Rev.

[4] Xiao C, Dash S, Morgantini C, Hegele RA, Lewis GF (2016). Pharmacological Targeting of the Atherogenic Dyslipidemia Complex: The Next Frontier in CVD Prevention Beyond Lowering LDL Cholesterol. Diabetes.

[5] Nordestgaard BG, Varbo A (2014). Triglycerides and cardiovascular disease. Lancet (London England).

[6] Group HTC, Landray MJ, Haynes R, Hopewell JC, Parish S, Aung T, Tomson J, Wallendszus K, Craig M, Jiang L (2014). Effects of extended-release niacin with laropiprant in high-risk patients. N Engl J Med.

[7] Lincoff AM, Nicholls SJ, Riesmeyer JS, Barter PJ, Brewer HB, Fox KAA, Gibson CM, Granger C, Menon V, Montalescot G (2017). Evacetrapib and Cardiovascular Outcomes in High-Risk Vascular Disease. N Engl J Med.

[8] Nordestgaard BG, Benn M, Schnohr P, Tybjaerg-Hansen A (2007). Nonfasting triglycerides and risk of myocardial infarction, ischemic heart disease, and death in men and women. JAMA.

[9] Quispe R, Martin SS, Michos ED, Lamba I, Blumenthal RS, Saeed A, Lima JAC, Puri R, Nomura SO, Tsai MY, et al: Remnant cholesterol predicts cardiovascular disease beyond LDL and ApoB: a primary prevention study. Eur Heart J 2021. 10.1093/eurheartj/ehab432 PMC8572557 34293083

[10] Castaner O, Pinto X, Subirana I, Amor AJ, Ros E, Hernaez A, Martinez-Gonzalez MA, Corella D, Salas-Salvado J, Estruch R (2020). Remnant Cholesterol, Not LDL Cholesterol, Is Associated With Incident Cardiovascular Disease. J Am Coll Cardiol.

[11] Varbo A, Benn M, Tybjaerg-Hansen A, Nordestgaard BG (2013). Elevated remnant cholesterol causes both low-grade inflammation and ischemic heart disease, whereas elevated low-density lipoprotein cholesterol causes ischemic heart disease without inflammation. Circulation.

[12] Kim HK, Song SO, Noh J, Jeong IK, Lee BW (2020). Data Configuration and Publication Trends for the Korean National Health Insurance and Health Insurance Review & Assessment Database. Diabetes Metab J.

[13] Kim JH, Moon JS, Byun SJ, Lee JH, Kang DR, Sung KC, Kim JY, Huh JH (2020). Fatty liver index and development of cardiovascular disease in Koreans without pre-existing myocardial infarction and ischemic stroke: a large population-based study. Cardiovasc Diabetol.

[14] Ko SH, Han K, Lee YH, Noh J, Park CY, Kim DJ, Jung CH, Lee KU, Ko KS (2018). TaskForce Team for the Diabetes Fact Sheet of the Korean Diabetes A: Past and Current Status of Adult Type 2 Diabetes Mellitus Management in Korea: A National Health Insurance Service Database Analysis. Diabetes Metab J.

[15] Ahn E, Shin DW, Yang HK, Yun JM, Chun SH, Suh B, Lee H, Son KY, Cho B (2015). Treatment Gap in the National Health-screening Program in Korea: Claim-based Follow-up of Statin Use for Sustained Hypercholesterolemia. J Korean Med Sci.

[16] Kim YH, Kang JG, Lee SJ, Han KD, Ihm SH, Cho KH, Park YG (2020). Underweight Increases the Risk of End-Stage Renal Diseases for Type 2 Diabetes in Korean Population: Data From the National Health Insurance Service Health Checkups 2009–2017. Diabetes Care.

[17] Kim BY, Kang SM, Kang JH, Kang SY, Kim KK, Kim KB, Kim B, Kim SJ, Kim YH, Kim JH (2021). 2020 Korean Society for the Study of Obesity Guidelines for the Management of Obesity in Korea. J Obes Metab Syndr.

[18] Kim MK, Han K, Kim HS, Park YM, Kwon HS, Yoon KH, Lee SH (2017). Cholesterol variability and the risk of mortality, myocardial infarction, and stroke: a nationwide population-based study. Eur Heart J.

[19] Sone H, Nakagami T, Nishimura R, Tajima N, Group MS (2016). Comparison of lipid parameters to predict cardiovascular events in Japanese mild-to-moderate hypercholesterolemic patients with and without type 2 diabetes: Subanalysis of the MEGA study. Diabetes Res Clin Pract.

[20] Emerging Risk Factors C, Di Angelantonio E, Sarwar N, Perry P, Kaptoge S, Ray KK, Thompson A, Wood AM, Lewington S, Sattar N (2009). Major lipids, apolipoproteins, and risk of vascular disease. JAMA.

[21] Ye X, Kong W, Zafar MI, Chen LL (2019). Serum triglycerides as a risk factor for cardiovascular diseases in type 2 diabetes mellitus: a systematic review and meta-analysis of prospective studies. Cardiovasc Diabetol.

[22] Redgrave TG, Snibson DA (1977). Clearance of chylomicron triacylglycerol and cholesteryl ester from the plasma of streptozotocin-induced diabetic and hypercholesterolemic hypothyroid rats. Metabolism.

[23] Proctor SD, Pabla CK, Mamo JC (2000). Arterial intimal retention of pro-atherogenic lipoproteins in insulin deficient rabbits and rats. Atherosclerosis.

[24] Mach F, Baigent C, Catapano AL, Koskinas KC, Casula M, Badimon L, Chapman MJ, De Backer GG, Delgado V, Ference BA (2020). 2019 ESC/EAS Guidelines for the management of dyslipidaemias: lipid modification to reduce cardiovascular risk. Eur Heart J.

[25] Johannesen CDL, Mortensen MB, Langsted A, Nordestgaard BG (2021). Apolipoprotein B and Non-HDL Cholesterol Better Reflect Residual Risk Than LDL Cholesterol in Statin-Treated Patients. J Am Coll Cardiol.

[26] Fox CS, Sullivan L, D’Agostino RB, Sr., Wilson PW, Framingham Heart S (2004). The significant effect of diabetes duration on coronary heart disease mortality: the Framingham Heart Study. Diabetes Care.

[27] Schaefer EJ, McNamara JR, Shah PK, Nakajima K, Cupples LA, Ordovas JM, Wilson PW, Framingham Offspring S (2002). Elevated remnant-like particle cholesterol and triglyceride levels in diabetic men and women in the Framingham Offspring Study. Diabetes Care.

[28] Tomono S, Kawazu S, Kato N, Ono T, Ishii C, Ito Y, Shimizu M, Shimoyama M, Nakano T, Nakajima K (1994). Uptake of remnant like particles (RLP) in diabetic patients from mouse peritoneal macrophages. J Atheroscler Thromb.

[29] Nordestgaard BG (2016). Triglyceride-Rich Lipoproteins and Atherosclerotic Cardiovascular Disease: New Insights From Epidemiology, Genetics, and Biology. Circ Res.

[30] Cao YX, Zhang HW, Jin JL, Liu HH, Zhang Y, Gao Y, Guo YL, Wu NQ, Hua Q, Li YF (2020). The longitudinal association of remnant cholesterol with cardiovascular outcomes in patients with diabetes and pre-diabetes. Cardiovasc Diabetol.

[31] Kim BY, Won JC, Lee JH, Kim HS, Park JH, Ha KH, Won KC, Kim DJ, Park KS (2019). Diabetes Fact Sheets in Korea, 2018: An Appraisal of Current Status. Diabetes Metab J.

