# Supplementary material for: Remnant cholesterol and the risk of cardiovascular disease in type 2 diabetes: a nationwide longitudinal cohort study
Source: Cardiovasc Diabetol. 2022 Nov 2;21:228. doi: 10.1186/s12933-022-01667-6 (PMC9632127; doi:10.1186/s12933-022-01667-6)
Supplement: Supplementary file 1 — Supplementary Material 1. Figure S1. Flowchart of study participants. Table S1. Association of baseline lipid values with incident myocardial infarction and stroke in patients with type 2 diabetes mellitus. Table S2. Hazard ratios and 95% confidence intervals of myocardial infarction (A) and ischemic stroke (B) according to the quartiles of the remnant cholesterol in subgroups. [file 12933_2022_1667_MOESM1_ESM.doc]

**Supporting information**

**Figure S1. Flowchart of study participants**


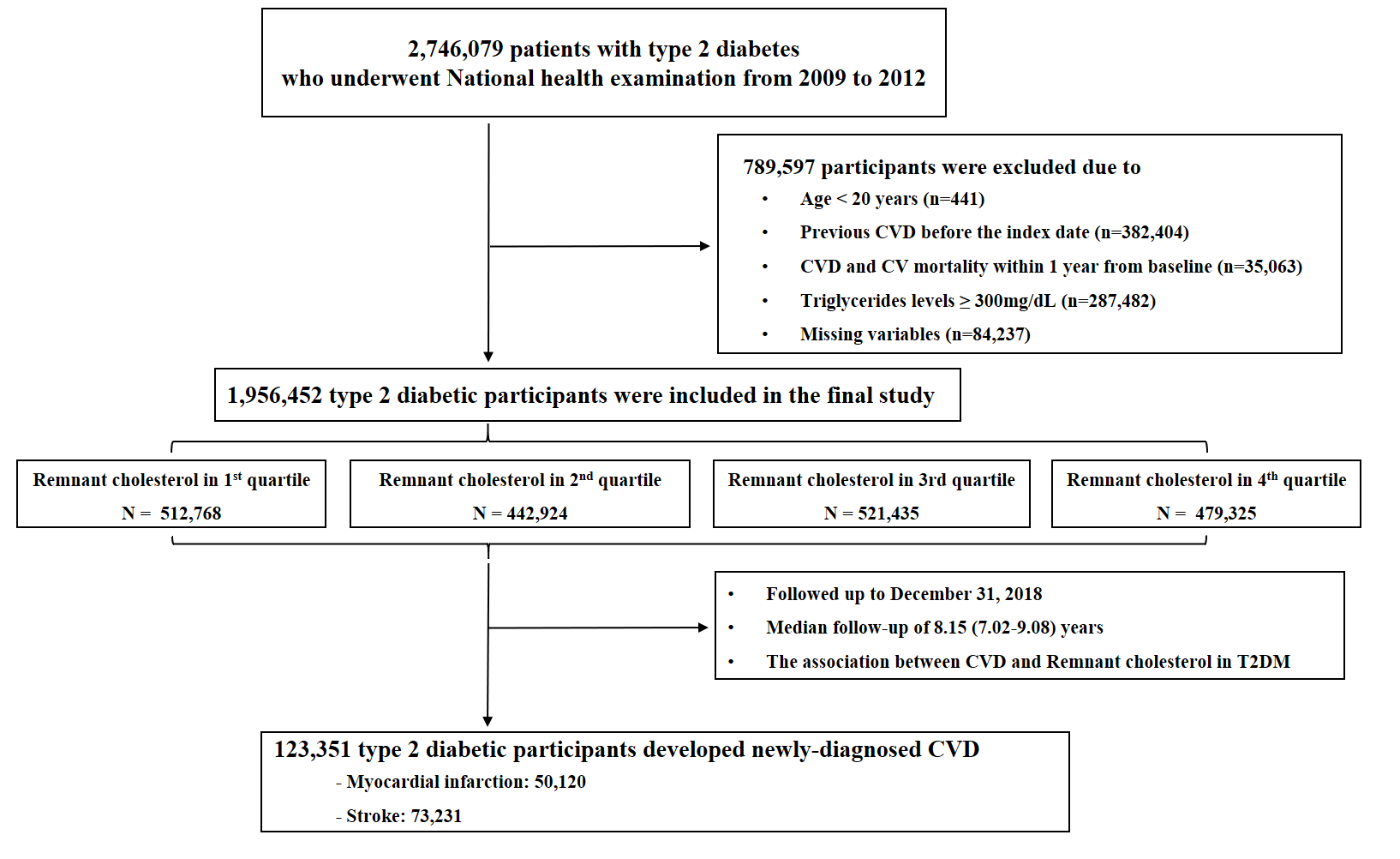


**Table S1.** Association of baseline lipid values with incident myocardial infarction and stroke in patients with type 2 diabetes mellitus

|  | **No event** | **Event** | **Hazard ratio* (95% CI)** | **P value** |
| --- | --- | --- | --- | --- |
| **Myocardial infarction** |  |  |  |  |
| **HDL cholesterol (mg/dL)** | 52.25±13.42 | 50.61±13.63 | +5mg: 0.977 (0.973,0.98) | <0.001 |
| **LDL cholesterol (mg/dL)** | 114.29±40.03 | 116.95±41.46 | +10mg/dL:1.014 (1.013,1.015) | <0.001 |
| **Triglyceride (mg/dL)** | 143.48±62.29 | 147.94±61.53 | +10mg/dL: 1.015 (1.013,1.016) | <0.001 |
| **Non-HDL cholesterol (mg/dL)** | 143.23±42.37 | 146.87±43.95 | +10mg/dL:1.016 (1.015,1.017) | <0.001 |
| **Remnant cholesterol (mg/dL)** | 28.93±13.4 | 29.92±13.42 | +10mg/dL:1.034(1.031,1.036) | <0.001 |
| **Stroke** |  |  |  |  |
| **HDL cholesterol (mg/dL)** | 52.25±13.41 | 51.07±13.66 | +5mg: 0.986(0.983,0.989) | <0.001 |
| **LDL cholesterol (mg/dL)** | 114.34±40.05 | 114.87±40.58 | +10mg/dL:1.01(1.009,1.011) | <0.001 |
| **Triglyceride (mg/dL)** | 143.49±62.32 | 146.29±61.06 | +10mg/dL:1.012(1.01,1.013) | <0.001 |
| **Non-HDL cholesterol (mg/dL)** | 143.28±42.4 | 144.4±42.89 | +10mg/dL:1.012(1.011,1.013) | <0.001 |
| **Remnant cholesterol (mg/dL)** | 28.94±13.42 | 29.53±12.75 | +10mg/dL:1.032(1.029,1.035) | <0.001 |

Values are mean ± SD. *Hazard ratios (HRs) were estimated by Cox proportional hazards regression models adjusted for age, sex, body mass index, smoking status, alcohol drinking status, regular exercise, low income, hypertension, statin treatment, fibrate use, duration of diabetes, and fasting blood glucose.

HDL, high-density lipoprotein; LDL, low-density lipoprotein; CI, confidence interval;

**Table S2. Hazard ratios and 95% confidence intervals of myocardial infarction (A) and ischemic stroke (B) according to the quartiles of the remnant cholesterol in subgroups**

1. **Myocardial infarction**

|  | **Quartiles** | **N** | **MI** | | | | |
| --- | --- | --- | --- | --- | --- | --- | --- |
|  |  |  | **EVENT** | **DURATION** | **RATE** | **HR(95% C.I)#** | **P for interactoin** |
| **Age <65** | Q1 | 366577 | 5476 | 2548219.5 | 2.14895 | 1(Reference) | **<.0001** |
|  | Q2 | 311574 | 5452 | 2172727.36 | 2.50929 | 1.117(1.076,1.16) |  |
|  | Q3 | 377903 | 7140 | 2634478.82 | 2.71021 | 1.205(1.163,1.248) |  |
|  | Q4 | 370496 | 7700 | 2581372.03 | 2.98291 | 1.356(1.309,1.404) |  |
| **Age ≥65** | Q1 | 146191 | 6004 | 956748.73 | 6.27542 | 1(Reference) |  |
|  | Q2 | 131350 | 5792 | 866673.46 | 6.68302 | 1.052(1.015,1.091) |  |
|  | Q3 | 143532 | 6974 | 952115.75 | 7.32474 | 1.15(1.111,1.191) |  |
|  | Q4 | 108829 | 5582 | 726097.24 | 7.68768 | 1.203(1.16,1.248) |  |
| **Male** | Q1 | 283065 | 6861 | 1910900.16 | 3.59045 | 1(Reference) | 0.3389 |
|  | Q2 | 250503 | 6375 | 1703649.28 | 3.74197 | 1.064(1.028,1.101) |  |
|  | Q3 | 311079 | 8153 | 2126281.64 | 3.83439 | 1.157(1.12,1.195) |  |
|  | Q4 | 315285 | 8208 | 2166488.51 | 3.78862 | 1.262(1.221,1.304) |  |
| **Female** | Q1 | 229703 | 4619 | 1594068.07 | 2.89762 | 1(Reference) |  |
|  | Q2 | 192421 | 4869 | 1335751.54 | 3.64514 | 1.111(1.067,1.156) |  |
|  | Q3 | 210356 | 5961 | 1460312.92 | 4.082 | 1.202(1.157,1.25) |  |
|  | Q4 | 164040 | 5074 | 1140980.76 | 4.44705 | 1.31(1.258,1.363) |  |
| **Obese (-)** | Q1 | 332992 | 7459 | 2264277.24 | 3.29421 | 1(Reference) | 0.0939 |
|  | Q2 | 240315 | 6392 | 1635064.47 | 3.90933 | 1.098(1.062,1.135) |  |
|  | Q3 | 252015 | 7369 | 1719569.9 | 4.28537 | 1.201(1.162,1.24) |  |
|  | Q4 | 206695 | 6271 | 1416744.66 | 4.42634 | 1.283(1.241,1.328) |  |
| **Obese (+)** | Q1 | 179776 | 4021 | 1240690.98 | 3.24094 | 1(Reference) |  |
|  | Q2 | 202609 | 4852 | 1404336.35 | 3.45501 | 1.05(1.007,1.095) |  |
|  | Q3 | 269420 | 6745 | 1867024.67 | 3.6127 | 1.128(1.085,1.173) |  |
|  | Q4 | 272630 | 7011 | 1890724.61 | 3.7081 | 1.252(1.204,1.302) |  |
| **Abdominal obesity (-)** | Q1 | 380998 | 8027 | 2602899 | 3.08387 | 1(Reference) | 0.1313 |
|  | Q2 | 286833 | 6824 | 1966326 | 3.47043 | 1.080(1.045,1.115) |  |
|  | Q3 | 311601 | 8050 | 2142398 | 3.75747 | 1.195(1.158,1.233) |  |
|  | Q4 | 268394 | 7070 | 1852449 | 3.81657 | 1.288(1.247,1.331) |  |
| **Abdominal obesity*(+)** | Q1 | 131770 | 3453 | 902068.8 | 3.82787 | 1(Reference) |  |
|  | Q2 | 156091 | 4420 | 1073074 | 4.11901 | 1.077(1.030,1.126) |  |
|  | Q3 | 209834 | 6064 | 1444197 | 4.19887 | 1.134(1.087,1.182) |  |
|  | Q4 | 210931 | 6212 | 1455020 | 4.26936 | 1.250(1.199,1.303) |  |
| **Hypertension (-)** | Q1 | 268373 | 4404 | 1847672.95 | 2.38354 | 1(Reference) | 0.0159 |
|  | Q2 | 205896 | 3798 | 1424142.21 | 2.66687 | 1.082(1.036,1.13) |  |
|  | Q3 | 232288 | 4664 | 1608685.05 | 2.89926 | 1.212(1.163,1.263) |  |
|  | Q4 | 212412 | 4420 | 1474590 | 2.99744 | 1.34(1.285,1.398) |  |
| **Hypertension (+)** | Q1 | 244395 | 7076 | 1657295.28 | 4.26961 | 1(Reference) |  |
|  | Q2 | 237028 | 7446 | 1615258.61 | 4.60979 | 1.082(1.047,1.117) |  |
|  | Q3 | 289147 | 9450 | 1977909.51 | 4.77777 | 1.155(1.12,1.191) |  |
|  | Q4 | 266913 | 8862 | 1832879.27 | 4.83502 | 1.25(1.211,1.29) |  |
| **CKD (-)** | Q1 | 468861 | 9718 | 3211554.95 | 3.02595 | 1(Reference) | 0.7382 |
|  | Q2 | 397615 | 9242 | 2736911.54 | 3.3768 | 1.082(1.051,1.113) |  |
|  | Q3 | 466122 | 11385 | 3216756.16 | 3.53928 | 1.162(1.131,1.194) |  |
|  | Q4 | 429885 | 10696 | 2975221.48 | 3.59503 | 1.262(1.227,1.298) |  |
| **CKD (+)** | Q1 | 43907 | 1762 | 293413.27 | 6.00518 | 1(Reference) |  |
|  | Q2 | 45309 | 2002 | 302489.27 | 6.61842 | 1.052(0.987,1.122) |  |
|  | Q3 | 55313 | 2729 | 369838.4 | 7.3789 | 1.169(1.101,1.242) |  |
|  | Q4 | 49440 | 2586 | 332247.79 | 7.78335 | 1.264(1.19,1.344) |  |
| **Statin (-)** | Q1 | 389915 | 8408 | 2655964.22 | 3.16571 | 1(Reference) | 0.0845 |
|  | Q2 | 325461 | 7943 | 2226181.21 | 3.56799 | 1.071(1.038,1.104) |  |
|  | Q3 | 382429 | 9884 | 2624752.94 | 3.76569 | 1.156(1.123,1.191) |  |
|  | Q4 | 355307 | 9347 | 2446874.79 | 3.81997 | 1.257(1.219,1.295) |  |
| **Statin (+)** | Q1 | 122853 | 3072 | 849004 | 3.61836 | 1(Reference) |  |
|  | Q2 | 117463 | 3301 | 813219.61 | 4.05917 | 1.116(1.063,1.173) |  |
|  | Q3 | 139006 | 4230 | 961841.62 | 4.39781 | 1.226(1.17,1.284) |  |
|  | Q4 | 124018 | 3935 | 860594.48 | 4.57242 | 1.346(1.283,1.411) |  |
| **Fibrate (-)** | Q1 | 502962 | 11206 | 3436238.98 | 3.26112 | 1(Reference) | 0.4287 |
|  | Q2 | 433247 | 10981 | 2971973.72 | 3.69485 | 1.085(1.057,1.114) |  |
|  | Q3 | 509077 | 13771 | 3500721.17 | 3.93376 | 1.178(1.149,1.208) |  |
|  | Q4 | 463054 | 12820 | 3193952.79 | 4.01384 | 1.285(1.252,1.319) |  |
| **Fibrate (+)** | Q1 | 9806 | 274 | 68729.25 | 3.98666 | 1(Reference) |  |
|  | Q2 | 9677 | 263 | 67427.1 | 3.90051 | 1.006(0.85,1.192) |  |
|  | Q3 | 12358 | 343 | 85873.39 | 3.99425 | 1.066(0.91,1.25) |  |
|  | Q4 | 16271 | 462 | 113516.48 | 4.06989 | 1.134(0.976,1.316) |  |
| **Metabolic Syndrome (-)** | Q1 | 282905 | 5432 | 1928926.08 | 2.81607 | 1(Reference) | 0.8411 |
|  | Q2 | 200038 | 4274 | 1369685.68 | 3.12042 | 1.072(1.03,1.116) |  |
|  | Q3 | 121796 | 2522 | 838005.02 | 3.00953 | 1.131(1.079,1.186) |  |
|  | Q4 | 46228 | 811 | 320455.79 | 2.53077 | 1.214(1.127,1.308) |  |
| **Metabolic Syndrome (+)** | Q1 | 229863 | 6048 | 1576042.14 | 3.83746 | 1(Reference) |  |
|  | Q2 | 242886 | 6970 | 1669715.13 | 4.17436 | 1.083(1.046,1.121) |  |
|  | Q3 | 399639 | 11592 | 2748589.54 | 4.21744 | 1.158(1.122,1.195) |  |
|  | Q4 | 433097 | 12471 | 2987013.48 | 4.17507 | 1.247(1.208,1.287) |  |
| **TG <150** | Q1 | 508492 | 11421 | 3476044.5 | 3.28563 | 1(Reference) | 0.1691 |
|  | Q2 | 437174 | 11128 | 2999944.6 | 3.7094 | 1.082(1.054,1.11) |  |
|  | Q3 | 205260 | 5471 | 1411118.25 | 3.87707 | 1.136(1.1,1.174) |  |
|  | Q4 | 4007 | 102 | 28721.47 | 3.55135 | 1.183(0.974,1.438) |  |
| **TG ≥150** | Q1 | 4276 | 59 | 28923.72 | 2.03985 | 1(Reference) |  |
|  | Q2 | 5750 | 116 | 39456.22 | 2.93997 | 1.363(0.997,1.865) |  |
|  | Q3 | 316175 | 8643 | 2175476.32 | 3.97292 | 1.511(1.169,1.952) |  |
|  | Q4 | 475318 | 13180 | 3278747.8 | 4.01983 | 1.613(1.249,2.083) |  |
| **HDL-C ≥40/50** | Q1 | 425704 | 9136 | 2911784.5 | 3.13759 | 1(Reference) | 0.117 |
|  | Q2 | 337670 | 8180 | 2319876.29 | 3.52605 | 1.092(1.06,1.125) |  |
|  | Q3 | 366646 | 9207 | 2525162.8 | 3.6461 | 1.169(1.135,1.204) |  |
|  | Q4 | 308700 | 7752 | 2131456.88 | 3.63695 | 1.268(1.23,1.308) |  |
| **HDL-C <40/50** | Q1 | 87064 | 2344 | 593183.73 | 3.95156 | 1(Reference) |  |
|  | Q2 | 105254 | 3064 | 719524.52 | 4.25837 | 1.017(0.964,1.074) |  |
|  | Q3 | 154789 | 4907 | 1061431.76 | 4.623 | 1.114(1.06,1.17) |  |
|  | Q4 | 170625 | 5530 | 1176012.39 | 4.70233 | 1.195(1.139,1.255) |  |

1. **Stroke**

|  | **Quartiles** | **N** | **Stroke** | | | | |
| --- | --- | --- | --- | --- | --- | --- | --- |
|  |  |  | **EVENT** | **DURATION** | **RATE** | **HR(95% C.I)** | **inter p.** |
| **Age <65** | Q1 | 366577 | 7049 | 2541373.26 | 2.7737 | 1(Reference) | 0.7083 |
|  | Q2 | 311574 | 6927 | 2166761.14 | 3.1969 | 1.098(1.062,1.135) |  |
|  | Q3 | 377903 | 8852 | 2627334.02 | 3.3692 | 1.15(1.114,1.187) |  |
|  | Q4 | 370496 | 9115 | 2575105.59 | 3.5397 | 1.231(1.193,1.271) |  |
| **Age ≥65** | Q1 | 146191 | 10094 | 943998.73 | 10.6928 | 1(Reference) |  |
|  | Q2 | 131350 | 9958 | 853484.71 | 11.6675 | 1.073(1.044,1.103) |  |
|  | Q3 | 143532 | 11711 | 936516.41 | 12.5049 | 1.146(1.116,1.177) |  |
|  | Q4 | 108829 | 9525 | 712687.89 | 13.3649 | 1.212(1.178,1.247) |  |
| **Male** | Q1 | 283065 | 10182 | 1899501.63 | 5.36035 | 1(Reference) | **0.0073** |
|  | Q2 | 250503 | 9562 | 1693401.68 | 5.64662 | 1.084(1.055,1.115) |  |
|  | Q3 | 311079 | 11631 | 2114505.92 | 5.50058 | 1.137(1.107,1.168) |  |
|  | Q4 | 315285 | 11045 | 2156567.51 | 5.12156 | 1.189(1.157,1.222) |  |
| **Female** | Q1 | 229703 | 6961 | 1585870.36 | 4.38939 | 1(Reference) |  |
|  | Q2 | 192421 | 7323 | 1326844.16 | 5.51911 | 1.082(1.047,1.118) |  |
|  | Q3 | 210356 | 8932 | 1449344.52 | 6.16279 | 1.161(1.125,1.198) |  |
|  | Q4 | 164040 | 7595 | 1131225.98 | 6.71395 | 1.267(1.226,1.309) |  |
| **Obese (-)** | Q1 | 332992 | 11314 | 2251046.89 | 5.02611 | 1(Reference) | **0.0005** |
|  | Q2 | 240315 | 9617 | 1624191.54 | 5.9211 | 1.072(1.043,1.101) |  |
|  | Q3 | 252015 | 11009 | 1707162.91 | 6.44871 | 1.166(1.135,1.197) |  |
|  | Q4 | 206695 | 9309 | 1405864.49 | 6.62155 | 1.246(1.212,1.281) |  |
| **Obese (+)** | Q1 | 179776 | 5829 | 1234325.1 | 4.72242 | 1(Reference) |  |
|  | Q2 | 202609 | 7268 | 1396054.3 | 5.2061 | 1.082(1.046,1.12) |  |
|  | Q3 | 269420 | 9554 | 1856687.53 | 5.14572 | 1.105(1.07,1.142) |  |
|  | Q4 | 272630 | 9331 | 1881928.99 | 4.95821 | 1.17(1.132,1.209) |  |
| **Abdominal obesity (-)** | Q1 | 380998 | 11841 | 2589691 | 4.57236 | 1(Reference) | 0.0034 |
|  | Q2 | 286833 | 10283 | 1954880 | 5.26017 | 1.095 (1.067, 1.125) |  |
|  | Q3 | 311601 | 11649 | 2129693 | 5.4698 | 1.172 (1.142, 1.202) |  |
|  | Q4 | 268394 | 9972 | 1841803 | 5.41426 | 1.247 (1.214, 1.281) |  |
| **Abdominal obesity* (+)** | Q1 | 131770 | 5302 | 895681 | 5.91952 | 1(Reference) |  |
|  | Q2 | 156091 | 6602 | 1065366 | 6.19693 | 1.047 (1.010, 1.086) |  |
|  | Q3 | 209834 | 8914 | 1434157 | 6.2155 | 1.092 (1.056, 1.130) |  |
|  | Q4 | 210931 | 8668 | 1445991 | 5.99451 | 1.160 (1.120, 1.200) |  |
| **Hypertension (-)** | Q1 | 268373 | 6024 | 1841590.04 | 3.27109 | 1(Reference) | 0.2834 |
|  | Q2 | 205896 | 5331 | 1418726.75 | 3.75759 | 1.113(1.073,1.155) |  |
|  | Q3 | 232288 | 6036 | 1603447.69 | 3.76439 | 1.163(1.122,1.206) |  |
|  | Q4 | 212412 | 5355 | 1470751.49 | 3.641 | 1.225(1.18,1.271) |  |
| **Hypertension (+)** | Q1 | 244395 | 11119 | 1643781.95 | 6.76428 | 1(Reference) |  |
|  | Q2 | 237028 | 11554 | 1601519.1 | 7.2144 | 1.068(1.041,1.096) |  |
|  | Q3 | 289147 | 14527 | 1960402.75 | 7.41021 | 1.139(1.111,1.168) |  |
|  | Q4 | 266913 | 13285 | 1817041.99 | 7.31133 | 1.217(1.186,1.248) |  |
| **CKD (-)** | Q1 | 468861 | 14520 | 3194997.3 | 4.5446 | 1(Reference) | 0.5942 |
|  | Q2 | 397615 | 13884 | 2720982.05 | 5.1026 | 1.085(1.06,1.111) |  |
|  | Q3 | 466122 | 16587 | 3198311.82 | 5.1862 | 1.14(1.115,1.166) |  |
|  | Q4 | 429885 | 14963 | 2959560.78 | 5.0558 | 1.21(1.182,1.238) |  |
| **CKD (+)** | Q1 | 43907 | 2623 | 290374.69 | 9.0332 | 1(Reference) |  |
|  | Q2 | 45309 | 3001 | 299263.79 | 10.0279 | 1.046(0.993,1.103) |  |
|  | Q3 | 55313 | 3976 | 365538.62 | 10.8771 | 1.132(1.078,1.19) |  |
|  | Q4 | 49440 | 3677 | 328232.7 | 11.2024 | 1.199(1.14,1.261) |  |
| **Statin (-)** | Q1 | 389915 | 12981 | 2640345 | 4.9164 | 1(Reference) | **<.0001** |
|  | Q2 | 325461 | 12432 | 2210840.46 | 5.6232 | 1.076(1.05,1.103) |  |
|  | Q3 | 382429 | 14899 | 2607146.71 | 5.71468 | 1.126(1.099,1.153) |  |
|  | Q4 | 355307 | 13458 | 2431453.63 | 5.53496 | 1.186(1.157,1.216) |  |
| **Statin (+)** | Q1 | 122853 | 4162 | 845026.99 | 4.92529 | 1(Reference) |  |
|  | Q2 | 117463 | 4453 | 809405.38 | 5.50157 | 1.106(1.06,1.154) |  |
|  | Q3 | 139006 | 5664 | 956703.73 | 5.92033 | 1.212(1.164,1.262) |  |
|  | Q4 | 124018 | 5182 | 856339.85 | 6.05134 | 1.322(1.269,1.378) |  |
| **Fibrate (-)** | Q1 | 502962 | 16813 | 3417055.76 | 4.92032 | 1(Reference) | 0.0594 |
|  | Q2 | 433247 | 16496 | 2953309.86 | 5.5856 | 1.08(1.057,1.103) |  |
|  | Q3 | 509077 | 20033 | 3478626.65 | 5.75888 | 1.142(1.119,1.166) |  |
|  | Q4 | 463054 | 17978 | 3175125.59 | 5.66214 | 1.218(1.192,1.244) |  |
| **Fibrate (+)** | Q1 | 9806 | 330 | 68316.23 | 4.83048 | 1(Reference) |  |
|  | Q2 | 9677 | 389 | 66935.99 | 5.81152 | 1.241(1.072,1.437) |  |
|  | Q3 | 12358 | 530 | 85223.79 | 6.21892 | 1.381(1.204,1.585) |  |
|  | Q4 | 16271 | 662 | 112667.89 | 5.87568 | 1.355(1.187,1.546) |  |
| **Metabolic Syndrome (-)** | Q1 | 282905 | 8298 | 1919209.47 | 4.32366 | 1(Reference) | 0.8448 |
|  | Q2 | 200038 | 6632 | 1361784.23 | 4.87008 | 1.085(1.05,1.12) |  |
|  | Q3 | 121796 | 3705 | 833634.95 | 4.44439 | 1.121(1.078,1.166) |  |
|  | Q4 | 46228 | 1081 | 319512.75 | 3.38328 | 1.208(1.133,1.288) |  |
| **Metabolic Syndrome (+)** | Q1 | 229863 | 8845 | 1566162.52 | 5.64756 | 1(Reference) |  |
|  | Q2 | 242886 | 10253 | 1658461.62 | 6.18224 | 1.077(1.047,1.108) |  |
|  | Q3 | 399639 | 16858 | 2730215.48 | 6.1746 | 1.137(1.107,1.167) |  |
|  | Q4 | 433097 | 17559 | 2968280.74 | 5.91555 | 1.199(1.168,1.23) |  |
| **TG <150** | Q1 | 508492 | 17041 | 3456588.61 | 4.93001 | 1(Reference) | 0.7584 |
|  | Q2 | 437174 | 16719 | 2980986.26 | 5.60855 | 1.082(1.06,1.106) |  |
|  | Q3 | 205260 | 8197 | 1401489.21 | 5.84878 | 1.137(1.108,1.168) |  |
|  | Q4 | 4007 | 138 | 28606.5 | 4.82408 | 1.102(0.932,1.303) |  |
| **TG ≥150** | Q1 | 4276 | 102 | 28783.38 | 3.54371 | 1(Reference) |  |
|  | Q2 | 5750 | 166 | 39259.58 | 4.22827 | 1.126(0.88,1.441) |  |
|  | Q3 | 316175 | 12366 | 2162361.22 | 5.71875 | 1.176(0.967,1.429) |  |
|  | Q4 | 475318 | 18502 | 3259186.98 | 5.67688 | 1.244(1.024,1.512) |  |
| **HDL ≥40/50** | Q1 | 425704 | 13723 | 2895909.92 | 4.73875 | 1(Reference) | 0.0578 |
|  | Q2 | 337670 | 12316 | 2305928.68 | 5.34102 | 1.091(1.065,1.118) |  |
|  | Q3 | 366646 | 13429 | 2510269.42 | 5.34962 | 1.142(1.115,1.169) |  |
|  | Q4 | 308700 | 10917 | 2120034.42 | 5.14944 | 1.216(1.185,1.248) |  |
| **HDL-C <40/50** | Q1 | 87064 | 3420 | 589462.07 | 5.8019 | 1(Reference) |  |
|  | Q2 | 105254 | 4569 | 714317.17 | 6.39632 | 1.026(0.982,1.073) |  |
|  | Q3 | 154789 | 7134 | 1053581.02 | 6.77119 | 1.1(1.055,1.145) |  |
|  | Q4 | 170625 | 7723 | 1167759.07 | 6.61352 | 1.147(1.101,1.194) |  |

^#^Adjusted model: Adjusted for age, sex, body mass index, smoking status, alcohol drinking status, regular exercise, low income, hypertension, statin use, fibrate use, duration of diabetes, and fasting blood glucose

*Abdominal obesity: waist circumference ≥ 90 cm for men or ≥ 85 cm for women

CKD, chronic kidney disease; MetS, metabolic syndrome; TG, triglyceride; HDL-C; high density lipoprotein cholesterol; CI, confidence interval; HR, Hazard ratio;
